# Adaptive Bi-Operator Evolution for Multitasking Optimization Problems

**DOI:** 10.3390/biomimetics9100604

**Published:** 2024-10-08

**Authors:** Changlong Wang, Zijia Wang, Zheng Kou

**Affiliations:** 1 School of Computer Science and Cyber Engineering, Guangzhou University, Guangzhou 510006, China; 2112306006@e.gzhu.edu.cn; 2 Institute of Computing Science and Technology, Guangzhou University, Guangzhou 510006, China

**Keywords:** evolutionary multitask optimization (EMTO), evolutionary computation (EC), evolutionary search operator, knowledge transfer

## Abstract

The field of evolutionary multitasking optimization (EMTO) has been a highly anticipated research topic in recent years. EMTO aims to utilize evolutionary algorithms to concurrently solve complex problems involving multiple tasks. Despite considerable advancements in this field, numerous evolutionary multitasking algorithms continue to use a single evolutionary search operator (ESO) throughout the evolution process. This strategy struggles to completely adapt to different tasks, consequently hindering the algorithm’s performance. To overcome this challenge, this paper proposes multitasking evolutionary algorithms via an adaptive bi-operator strategy (BOMTEA). BOMTEA adopts a bi-operator strategy and adaptively controls the selection probability of each ESO according to its performance, which can determine the most suitable ESO for various tasks. In an experiment, BOMTEA showed outstanding results on two well-known multitasking benchmark tests, CEC17 and CEC22, and significantly outperformed other comparative algorithms.

## 1. Introduction

Evolutionary computation (EC) algorithms, including the genetic algorithm (GA) [1,2,3], particle swarm optimization (PSO) [4,5,6], and differential evolution (DE) [7,8,9], represent a type of heuristic optimization algorithm based on biological evolution process [10,11] and are suitable for various optimization problems, such as multimodal [12,13,14], large-scale [15,16], and multi-objective problems [17,18]. Because of its simplicity and ease of implementation, EC has found widespread applications across various fields, such as engineering [19,20], economics [21,22], biology [23], and computer science [24,25].

The traditional EC algorithm was originally designed to solve a single optimization problem or independent optimization problems. However, many optimization problems in the real world are interconnected or exhibit similarities to each other [26,27,28]. In this case, evolutionary multitasking optimization (EMTO) has emerged as a new research topic in EC, aiming to solve multiple problems simultaneously [29].

Generally, many multitasking evolutionary algorithms (MTEAs) only use one evolutionary search operator (ESO). For example, the multifactorial evolutionary algorithm (MFEA) [30], multifactorial evolutionary algorithm with online transfer parameter estimation (MFEA-II) [31] and multitasking genetic algorithm (MTGA) [32] only use the GA. Multifactorial DE (MFDE) [33], domain adaptation multitask optimization (DAMTO) [34] and block-level knowledge transfer DE (BLKT-DE) [35] only use DE/rand/1 to generate offspring. However, it is well known that only one ESO is not suitable for all questions. Taking the widely used CEC17 MTO benchmarks [36] as an example, according to the originally reported results of MFDE [33] and MFEA [30], MFDE performs better than MFEA on the complete-intersection, high-similarity (CIHS) and complete-intersection, medium-similarity (CIMS) problems, which indicates that the DE/rand/1 operator is suitable for the CIHS and CIMS problems. However, MFEA outperforms MFDE on the complete-intersection, low-similarity (CILS) problem, which means that the GA is more appropriate when solving CILS problems.

Therefore, some scholars began to use multiple ESOs to solve multitask problems. Feng et al. [37] introduced an EMT algorithm that features explicit genetic transfer between tasks (EMEA), where two populations are evolved using the GA and DE, respectively. Evolutionary multitasking via reinforcement learning (RLMFEA) [38] randomly selects DE or GA for evolution in each generation. Both EMEA and RLMFEA achieve better results than the previous algorithms, which indicates that the integration of the GA and DE has a good effect. However, this combination only remains at the fixed or random stage, without any adaptive mechanism. If the suitable ESO can be adaptively chosen for the corresponding problem, the performance will be significantly improved.

In this paper, an innovative MTEA called MTEA via adaptive bi-operator (BOMTEA) is proposed, which also combines the superiority of the GA and DE. Unlike other algorithms, the selection probability of each ESO is adaptively adjusted according to its performance, which can help the algorithm find the most suitable ESO for various tasks. In addition, MTEA also includes a novel knowledge transfer strategy to promote information sharing and communication among different tasks. The experimental results on multitasking benchmarks CEC17 and CEC22 fully demonstrate the effectiveness of BOMTEA, which can significantly outperform other comparative algorithms.

The rest of this article is structured as follows. Related knowledge of ESOs and the works related to EMTO are discussed in Section 2. BOMTEA is described in Section 3. Section 4 presents the experimental studies, while Section 5 concludes the article.

## 2. Preliminary

### 2.1. DE

The differential evolution (DE) algorithm seeks optimal solutions by simulating the evolutionary process observed in biological populations in nature. In DE, each potential solution is an individual, forming a population. These individuals improve and adapt to the environment through mutation, crossover, and selection operations.

1. Mutation

In DE, common mutation operations are based on differential mutation strategies. Taking the common DE/rand/1 as an example, for each individual (***x****_i_*), a new mutated individual (***v****_i_*) is generated according to Equation (1).

***v****_i_* = ***x****_r_*_1_ + *F* × (***x****_r_*_2_ − ***x****_r_*_3_) (1)

Here, *F* represents the scaling factor, while ***x****_r_*_1_, ***x****_r_*_2_, and ***x****_r_*_3_ are the different individuals randomly chosen from the population.

2. Crossover

Once the mutated individual (***v****_i_*) is generated, DE executes a crossover operation between ***v****_i_* and ***x****_i_* to produce the trial vector (***u****_i_*), as shown in Equation (2).

(2) $$u_{i,j}=\left\{\begin{array}{l} v_{i,j},\;\mathrm{if}\;\mathrm{rand}(0,\;1)\leq Cr\;\mathrm{or}\;j==j_{\mathrm{rand}}\\ x_{i,j},\;\mathrm{otherwise} \end{array}\right.$$

Here, *Cr* denotes the crossover rate. *j*_rand_ is a random integer within [1, D], ensuring that ***u****_i_* differs from ***x****_i_* in at least one dimension.

3. Selection

This operation involves choosing the superior individual between ***x****_i_* and ***u****_i_* for the next generation. When tackling a minimization problem, it can be represented as Equation (3).

(3) $$x_{i}=\left\{\begin{array}{l} u_{i},\;\mathrm{if}\;f(u_{i})\leq f(x_{i})\\ x_{i},\;\mathrm{otherwise} \end{array}\right.$$

where *f*(*) is the function value corresponding to the individual.

### 2.2. Simulated Binary Crossover (SBX)

SBX is a crossover operation commonly used in evolutionary algorithms, especially in genetic algorithms. SBX is based on the exponential probability distribution, implying that the element at position *i* of the offspring is generated from an exponential probability distribution.

(4) $$c_{1}^{i}=\frac{1}{2}\cdot \left[\left(1-\beta \right)\cdot p_{1}^{i}+\left(1+\beta \right)\cdot p_{2}^{i}\right]\;c_{2}^{i}=\frac{1}{2}\cdot \left[\left(1+\beta \right)\cdot p_{1}^{i}+\left(1-\beta \right)\cdot p_{2}^{i}\right]$$

where *p*_1_ and *p*_2_ represent two different individuals of the parent and *c*_1_ and *c*_2_ represent two different individuals of the offspring. The distribution of *β* is specified by

(5) $$\beta (u)=\left\{\begin{array}{c} {\left(2u\right)}^{1/(\eta_{c}+1)},u\leq \frac{1}{2}\\ {\left[2(1-u)\right]}^{-1/(\eta_{c}+1)},u>\frac{1}{2} \end{array}\right.$$

where *u* is a randomly generated number within the range of [0, 1].

### 2.3. Evolutionary Multitasking Optimization

EMTO seeks to address multiple problems concurrently. EMTO can effectively utilize the correlation between tasks, so it can improve the results.

Suppose that EMTO comprises K single-objective tasks, all of which are minimization problems. The *i*th task, denoted as *T_i_*, encompasses a search space (*X_i_*) and an objective function (*F_i_*: *X_i_*→R) [33,39]. EMTO aims to discover a set of solutions that fulfill Equation (6).

(6) $$\left\{\left.x_{1}^{*},\;x_{2}^{*},\ldots ,\;x_{K}^{*}\right\}=\arg\min\{F_{1}(x_{1}),\;F_{2}(x_{2}),\ldots ,\;F_{K}(x_{K})\}\right.$$

### 2.4. MFEA

MFEA [30] is inspired by the biocultural models of multifactorial inheritance. Each individual in MFEA optimizes corresponding tasks through the skill factor. The framework of MFEA is shown in Algorithm 1, which includes assortative mating and vertical cultural transmission. When two individuals possessing different skill factors undergo a crossover operation with a random mating probability (*rmp*), information exchange between tasks occurs.
**Algorithm 1:** MFEA**Input: *p***_a_, ***p***_b_: two parent candidates randomly selected from ***pop***.**Output: *c***_a_, ***c***_b_: the offspring generated.**Begin**
1:  **If** τ_a_ == τ_b_ or *rand* < *rmp*:

2:    ***p****_a_* and ***p****_b_* crossover and mutate to get ***c**_a_* and ***c**_b_*.

3:       **If** *τ*_a_ == *τ*_b_:

4:         ***c***_a_ imitates ***p****_a_. **c***_b_ imitates ***p***_b_.

5:       **Else**

6:         **If** *rand* < 0.5:

7:           ***c***_a_ imitates ***p***_a_*. **c***_b_ imitates ***p***_b_.

8:         **Else**

9:           ***c***_a_ imitates ***p***_b_*. **c***_b_ imitates ***p***_a_.

10:         **End If**

11:       **End If**

12:  **Else**

13:    ***p***_a_ undergoes polynomial mutation to produce offspring **c**_a_.

14:    ***p****_b_* undergoes polynomial mutation to produce offspring ***c***_b_.
15:  ***c***_a_ imitates ***p***_a_*. **c***_b_ imitates ***p***_b_.

16:  **End If**
**End**

### 2.5. Related Work

In the past, evolutionary algorithms typically focused on solving individual optimization problems, but population-based search had implicit parallelism. Therefore, Gupta et al. [30] proposed a new framework for evolutionary multitasking (EMT) called MFEA. In this algorithm, complex developmental features are transmitted to offspring through the interaction of genetic and cultural factors. Later, many scholars conducted research on the following three aspects: “how to transfer”, “when to transfer”, and “what to transfer”.

For the first aspect of “how to transfer”, researchers have focused on methods to transfer data from the source task to the target task. For instance, Feng et al. [37] introduced an EMT algorithm that features explicit genetic transfer between tasks. According to this algorithm, the source task is the result of adding noise to the target task, so a denoising autoencoder is used to obtain the mapping from the source task to the target task. Wu and Tan [32] estimated the bias between the two tasks and removed it in chromosomal transfer to the solutions from different tasks can be closer to each other. Zhou et al. [40] achieved adaptive selection of crossover operators. Algorithm can select suitable crossover operators for different problems. Wang et al. [34] used transfer component analysis (TCA) to map two populations with different marginal probability distributions to the same space for knowledge transfer.

As the second aspect of “when to transfer”, the *rmp* determines the frequency of knowledge transfer between different tasks. In some literature [30,33], *rmp* used fixed values. Some researchers also attempted to make algorithms adaptively adjust *rmp* during operation. Liaw and Ting [41] introduced a framework known as the evolution of biocoenosis through symbiosis (EBS). EBS can control the information exchange frequency based on the times where the best solution of the current task is improved by other tasks or current task. In EMEA [37], knowledge transfer between tasks occurs at fixed intervals. Bali et al. [31] produced a framework that leverages the similarities and discrepancies among different tasks to enhance the optimization process. Li et al. [38] introduced a MFEA based on reinforcement learning, known as RLMFEA, which can adaptively adjust the *rmp* of different tasks. In DAMTO [34], when the fitness of this generation does not improve compared to the previous generation, knowledge transfer occurs.

As the three aspects of “what to transfer”, a simple method is to directly use the promising solutions of another task as knowledge [30,31,38,41]. In addition, there was some algorithms [32,34,37] that measure the differences between solutions from different tasks and take corresponding compensation measures to generate knowledge.

## 3. BOMTEA

In this section, the detailed information about the BOMTEA is proposed. Specifically, the motivation of BOMTEA is presented. Next, the detailed process for adaptively adjusting *eop* to control the selection of the two ESOs is given. Then, the method of knowledge transfer is developed. Finally, the complete BOMTEA algorithm is presented.

### 3.1. Motivation

In recent years, with the development of MTEAs, researchers have conducted in-depth explorations in these three aspects. Despite significant progress in MTEAs, many existing MTEAs still rely on a single ESO. For instance, algorithms such as MFEA [30], MFEA-II [31] and MTGA [32] exclusively use GA as their primary search strategy. Similarly, algorithms like MFDE [33], DAMTO [34] and BLKT-DE [35] rely solely on the DE/rand/1 strategy from DE to generate offspring. Each ESO has its unique characteristics and advantages. For example, DE is renowned for its rapid convergence speed, allowing it to quickly identify potentially excellent solutions. However, this fast convergence also makes DE more susceptible to getting trapped in local optima, which limits its performance in complex optimization problems. In contrast, the strength of GA lies in their ability to effectively maintain population diversity, providing strong exploratory capabilities in global search. Therefore, a single ESO is not suitable for all types of problems, as the nature and structure of different problems vary, leading to differing demands on search strategies.

Therefore, some scholars have begun to explore the use of multiple ESOs to solve multi-task optimization problems, aiming to improve the overall performance and adaptability of the algorithms. Feng et al. [37] proposed EMEA, which achieves intra-population evolution by assigning GA and DE to two different populations when handling multi-task problems. Another approach is RLMFEA [38], which randomly selects either DE or GA for each iteration. However, while this integration based on a random mechanism is effective, it still has certain limitations, as it cannot adaptively choose the most suitable ESO based on the specific characteristics of the problem. If the algorithm can adaptively select the appropriate ESO according to the specific problem, its performance will be significantly enhanced.

Therefore, this paper proposes an innovative MTEA called BOMTEA, which combines the advantages of GA and DE. The innovation of BOMTEA lies in its adaptive mechanism, which monitors the performance of each ESO and dynamically adjusts their selection probabilities. This approach allows for the effective utilization of the characteristics of GA and DE in different optimization environments, thereby achieving improved multi-task processing capabilities.

### 3.2. The Adaptive Bi-Operator Strategy

Specifically, if there are more offspring generated using DE that are retained to the next generation, then the next *eop* should be larger. On the contrary, if there are more offspring generated using GA that are retained to the next generation, the next *eop* should be smaller. The formula for *eop* is shown in Equation (7).

(7) $$eop_{i}^{t+1}=\frac{n_{i,DE}^{t}}{n_{i,DE}^{t}+n_{i,GA}^{t}}$$

where *eop_i_^t^*^+1^ is the *eop* of the task*_i_*, generation *t* + 1. *n^t^_i_*_,DE_ is a descendant generated in the *t*-th generation using the DE operator and retained for the next generation. *n^t^_i_*_,GA_ is a descendant using the GA operator and retained for the next generation.

Considering that a high *eop* (*eop* > 0.9) would cause the algorithm to rely almost entirely on DE, this could lead to premature convergence to a local optimum, resulting in a loss of population diversity and impacting the algorithm’s performance. On the other hand, a low *eop* (*eop* < 0.1) means that the algorithm would hardly use DE, thus losing the advantages that DE provides, which could significantly reduce search efficiency and affect the optimization results. Additionally, to emphasize the faster convergence speed of DE compared to GA, BOMTEA is inclined to use DE. Therefore, in this article, the range of *eop* is set to [0.3, 0.9].

The ESO used in the population evolution process is randomly assigned. As shown in Algorithm 2, the individual generates offspring via DE/rand/1 with the probability of *eop*. Otherwise, SBX and polynomial mutation are employed to generate offspring.
**Algorithm 2:** Adaptive Bi-operator Strategy**Input: *p***: a parent from target task.   *eop_i_*: Random selection probability of ESOs.**Output: *c***: the offspring generated.**Begin**
1:  **If**
*rand* < *eop_i_*:

2:    Generate offspring ***c*** using DE/rand/1.

3:  **Else**

4:    Generate offspring ***c*** using GA.

5:  **End If**
**End**

### 3.3. Knowledge Transfer

In EMTO, knowledge transfer plays a crucial role. It enables algorithms to share information across different tasks, thereby enhancing overall optimization efficiency. When knowledge gained from one task can be effectively applied to another task, the algorithm can accelerate the convergence process.

In order to perform knowledge transfer, if the ESO is GA, for each individual in the target task, an individual in the source task will be randomly selected for crossover and mutation, which are SBX and polynomial mutation respectively. Note that after crossover and mutation, two individuals are generated, but only one can participate in the evaluation. As shown in Algorithm 3, the selection here is random.

In order to perform knowledge transfer, if the ESO is DE, for each individual in the target task, an individual in the target task and two individuals in the source task will be randomly selected for DE. The special method is shown in Algorithm 4.
**Algorithm 3:** Knowledge Transfer of GA**Input: *p****_a_*: a parent from target task.   ***p****_b_*: a parent randomly selected from source task.**Output: *c***: the offspring generated.**Begin**
1:  **p**_a_ and ***p****_b_* crossover and mutate to give offspring ***c****_a_* and ***c****_b_*.

2:  **If** *rand* < 0.5:

3:    ***c*** = ***c****_a_*

4:  **Else**

5:    ***c*** = ***c****_b_*

6:  **End If**
**End**

**Algorithm 4:** Knowledge Transfer of DE**Input: *p****_t_*: a parent from target task.   ***pop****_t_*: the population of target task.   ***pop****_s_*: the population of source task.**Output: *c***: the offspring generated.
**Begin**

1:  Select one individual ***x***_r1_ from ***pop****_t_* randomly and ***x***_r1_! = ***p****_t_*.

2:  Select two individuals ***x***_r2_, ***x***_r3_ from ***pop****_s_* randomly and ***x***_r2_! = ***x***_r3_.

3:  According to Formula (1) to generate mutated individual ***v***_i_.

4:  According to Formula (2) to generate offspring ***c***.

**End**


### 3.4. Framework

The structure of BOMTEA for addressing two-task optimization problems is outlined in Algorithm 5. According to this algorithm, the primary steps of BOMTEA can be elucidated as follows:

Step 1: In a unified search space, the populations of two tasks are randomly initialized. Each individual’s dimension is set to the maximum value among the dimensions in all tasks. All dimensions of the individuals are normalized to [0, 1] according to the lower bound *L* and upper bound *U* of the solution space of task *T_j_*. This encoding method helps standardize the processing of features for different tasks. When the individual need to be evaluated, it should be decoded into the original solution by Equation (8) (line 1).

*x_i_* = *L_i_* + (*U_i_* − *L_i_*) × *y_i_* (8)

where *L_i_* is the minimum value of the *i*-th dimension of task *T_j_*, *U_i_* is the maximum value of the *i*-th dimension of task *T_j_*, *y_i_* is the normalized value of the *i*-th dimension of the individual and *x_i_* is the decoded value of the *i*-th dimension of the individual.

Step 2: Compute the fitness value of each individual of ***pop***_1_ and ***pop***_2_ (line 2).

Step 3: Each individual can be assigned an ESO via Algorithm 2 (line 5).

Step 4: Each individual from *task_i_* can perform knowledge transfer via with a probability of *rmp_i_*. The specific knowledge transfer methods are shown in Algorithms 3 and 4. If the ESO assigned to this individual is GA, then knowledge transfer is performed through Algorithm 3. Otherwise, knowledge transfer will be carried out through Algorithm 4. (line 6–7).

Step 5: Individuals who do not undergo knowledge transfer generate offspring via the ESO assigned. (line 9).

Step 6: Based on the fitness value, the elite selection strategy is employed to choose the most suitable individuals for constructing the subsequent generation population (line 12).

Step 7: Finally, in order to achieve adaptive adjustment of *eop* during iteration. Formula (7) is used to adjust the value of *eop* for each generation (line 13).
**Algorithm 5:** BOMTEA**Begin**
1:  Randomly initialize ***pop***_1_ and ***pop***_2_ for two tasks respectively. 2:  Evaluate each individual on each optimization task.

3:  **While** *FEs* < *maxFEs*:

4:    **For** each individual from ***pop***_1_ or ***pop***_2_:

5:      Perform **Algorithm 2** to allocate ESO.

6:      **If** *rand* < *rmp*_i_: 7:        Perform knowledge transfer via **Algorithm 3** or **4**.

8:      **Else**

9:        Generate offspring via ESO.

10:      **End If**

11:    **End For**

12:    Select the fittest individuals to form the next ***pop***_1_ or ***pop***_2_.

13:    Get new *eop*_1_ and *eop*_2_ via the Formula (7).

14:  **End While**
**End**

## 4. Experimental Studies

In this study, two widely used MTO benchmarks, CEC17 [36] and CEC22 [42] are chosen to evaluate the performance of the BOMTEA. Meanwhile, five MTEAs are used to be compared which are MFEA (2016) [30], EMEA (2019) [37], MFEA-AKT (2021) [40], MTGA (2020) [32] and RLMFEA (2023) [38]. There algorithms cover both single population algorithms and multiple population algorithms, spanning the time period from 2016 to 2023, so the experimental results are credible.

### 4.1. Experimental Setup

The relevant parameter settings for the algorithms involved are as follows.

- SBX and polynomial mutation in MFEA, EMEA, MFEA-AKT, MTGA, RLMFEA and BOMTEA: *η_c_* = 10, *η_m_* = 5.
- DE in EMEA, RLMFEA, BOMTEA: *F* = 0.5, *Cr* = 0.6.
- The random mating probability: *rmp* = 0.3.
- The initial random selection probability of ESO: *eop* = 0.5.
- Population size: *NP* = 100 for MFEA, EMEA, MFEA-AKT, MTGA, RLMFEA and BOMTEA.
- Maximum number of fitness evaluations: *MaxFEs* = 100,000.
- The parameters for which values are not provided are set to the optimal settings specified in the respective papers.

Each algorithm undergoes 30 independent runs to acquire the experimental outcomes. To assess the experimental results statistically, Wilcoxon’s rank sum test [43] with a significance level of α = 0.05 is employed.

The Origin 2022 version 9.9 is used to conduct the Wilcoxon’s rank sum test. In Origin, when selecting the Wilcoxon’s rank sum test, three null hypotheses *H*_0_ and alternative hypotheses *H*_1_ can be chosen:

(1) *H*_0_: Median_1_ = Median_2_ (If this null hypothesis is accepted, it indicates that our proposed algorithm and the comparison algorithm do not have a significant difference on this task).

*H*_1_: Median_1_ < > Median_2_ (If this alternative hypothesis is accepted, it indicates that our proposed algorithm and the comparison algorithm have significant difference on this task).

(2) *H*_0_: Median_1_ >= Median_2_.

*H*_1_: Median_1_ < Median_2_ (For minimization problems, if this alternative hypothesis is accepted, it indicates that our proposed algorithm performs significantly better than the comparison algorithm on this task).

(3) *H*_0_: Median_1_ <= Median_2_.

*H*_1_: Median_1_ > Median_2_ (For minimization problems, if this alternative hypothesis is accepted, it indicates that our proposed algorithm performs significantly worse than the comparison algorithm on this task).

By independently running the algorithm 30 times and testing with different alternative hypotheses, we can ultimately conclude that the results of our algorithm are “better/worse/similar to the results of the comparison algorithm. At the same time, the symbols “+/−/≈” denote that the results achieved by BOMTEA are “better/worse/similar to” those of the compared algorithm.

### 4.2. Experimental Results Comparisons on MTO Benchmarks

The mean fitness experimental results attained by BOMTEA, MFEA, EMEA, MFEA-AKT, MTGA and RLMFEA on CEC17 and CEC22 benchmarks are presented in Table 1. The best experimental result for each task is highlighted in boldface.

#### 4.2.1. CEC17 MTO Benchmarks

The CEC17 MTO benchmarks include nine benchmark problems, each containing two tasks. The problems can be divided into complete intersections (CI), partial intersections (PI), and no intersections (NI) based on their degree of intersection. They can also be divided into high similarity (HS), medium similarity (MS), and low similarity (LS) based on their similarity. Each task corresponds to an optimization function that needs to be optimized. For instance, in the CIHS problem, there are actually two tasks involved, making CIHS a two-task optimization problem. The functions that require optimization in this case are the Griewank function and the Rastrigin function. For more details, please refer to [36].

From Table 1, it can be seen that on the CEC17 benchmarks, BOMTEA consistently outperforms the compared algorithms. For the 9 total problems, for task 1, BOMTEA outperforms MFEA, EMEA, MFEA-AKT, MTGA, and RLMFEA on 9, 9, 9, 7 and 8 tasks, respectively, and is only worse on 1 task on the MTGA. For task 2, BOMTEA outperforms them on 7, 7, 7, 6 and 5 tasks, respectively. This suggests that when addressing MTO problems, the adaptive bi-operator strategy proves superior to the single-operator strategy.

**Figure 1:**
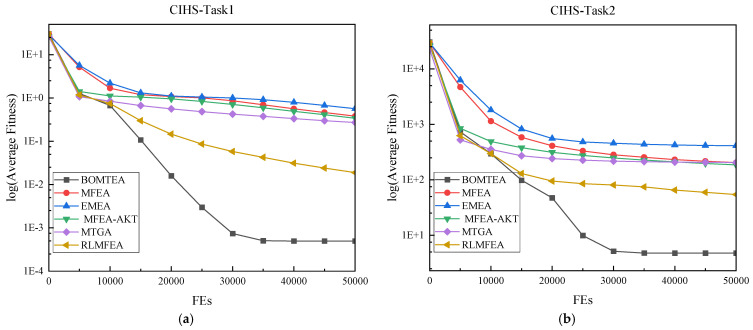

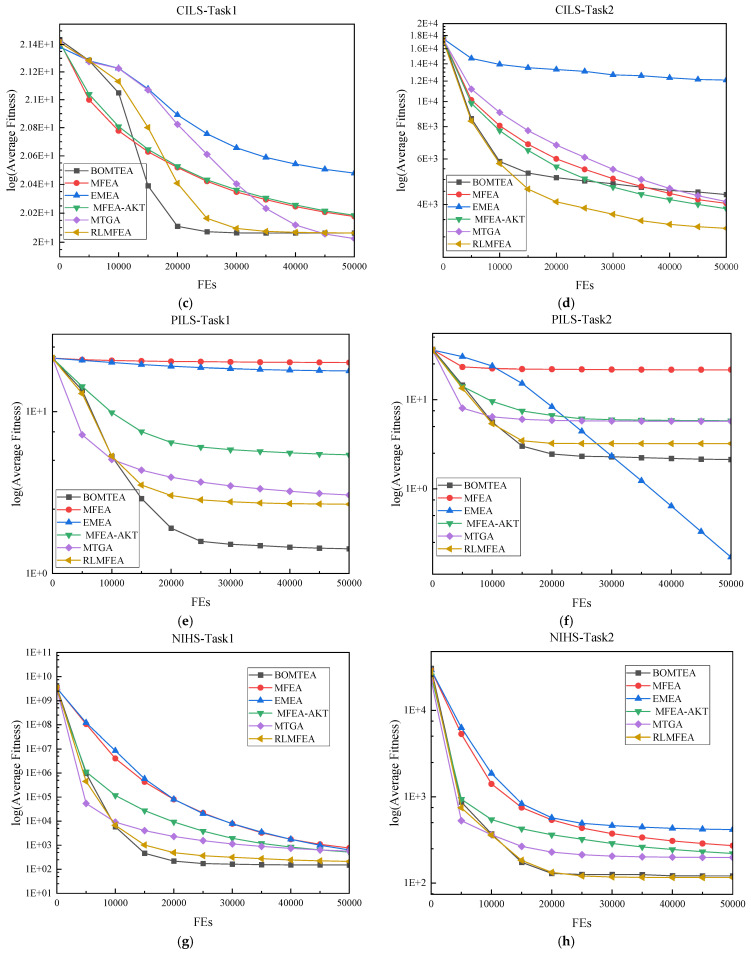
Convergence curves of the average fitness on (**a**) T1 of CEC17-CIHS; (**b**) T2 of CEC17-CIHS; (**c**) T1 of CEC17-CILS; (**d**) T2 of CEC17-CILS; (**e**) T1 of CEC17-PILS; (**f**) T2 of CEC17- PILS; (**g**) T1 of CEC17-NIHS; (**h**) T2 of CEC17-NIHS.

Furthermore, the convergence curves of BOMTEA and other comparative algorithms on the CEC17 problem are presented to study their convergence behavior, as shown in Figure 1. Firstly, as shown in Figure 1a–c,e, BOMTEA has a fast convergence rate on these issues. Secondly, as shown in Figure 1g,h, although the convergence speed of BOMTEA on this problem is almost the same as that of MTGA and RLMFEA in the early stage, the final results obtained by BOMTEA are superior to or are similar to these two algorithms respectively. Finally, as shown in Figure 1d, BOMTEA can easily get trapped in local optima when solving CILS problem. In practice, the comparative algorithms, including MFEA, EMEA, MFEA-AKT, and MTGA, also fall into local optima. However, BOMTEA has achieved significantly better results on other problems, further demonstrating the superiority of our proposed BOMTEA algorithm. Similarly, as shown in Figure 1f, the convergence speed of BOMTEA is slightly lower than that of EMEA but clearly faster than that of the other comparative algorithms. This indicates that, while there may be some discrepancies in certain specific scenarios, overall, BOMTEA exhibits strong performance and adaptability in solving various optimization problems, further reinforcing its position as an effective optimization algorithm. Note that GA and DE ESOs are also used in RLMFEA, but they are randomly selected and do not control their frequency. Therefore, although RLMFEA has similar results to BOMTEA in some problems, such as CILS, NIHS, NIMS, the overall results are not as good as BOMTEA.

#### 4.2.2. CEC22 MTO Benchmarks

Similarly, in Wilcoxon’s rank-sum test results on the CEC22 benchmarks, BOMTEA is generally superior to the compared algorithms. For the 10 total problems, for task 1 in MFEA, EMEA, MFEA-AKT, MTGA, and RLMFEA, BOMTEA is only worse on 0, 1, 0, 3 and 1 tasks respectively and outperforms on 9, 8, 5, 5 and 2 tasks. Compared to RLMFEA, BOMTEA has fewer dominant problems and most of them are similar. This may be because they all use bi-operator strategy. For task 2, BOMTEA outperforms MFEA, EMEA, MFEA-AKT, MTGA, and RLMFEA on 9, 9, 7, 5 and 3 tasks, respectively. This suggests that when tackling MTO problems, the adaptive bi-operator strategy proves superior to the single-operator strategy.

**Figure 2:**
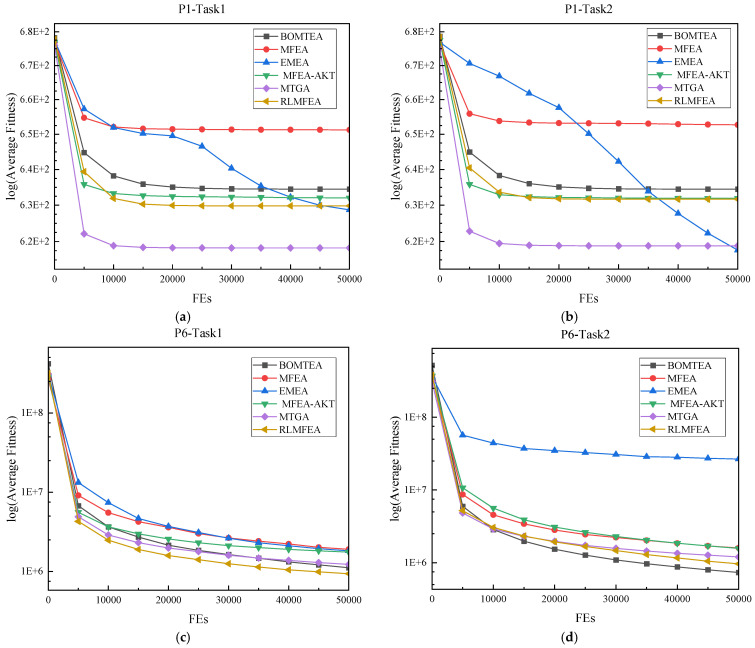

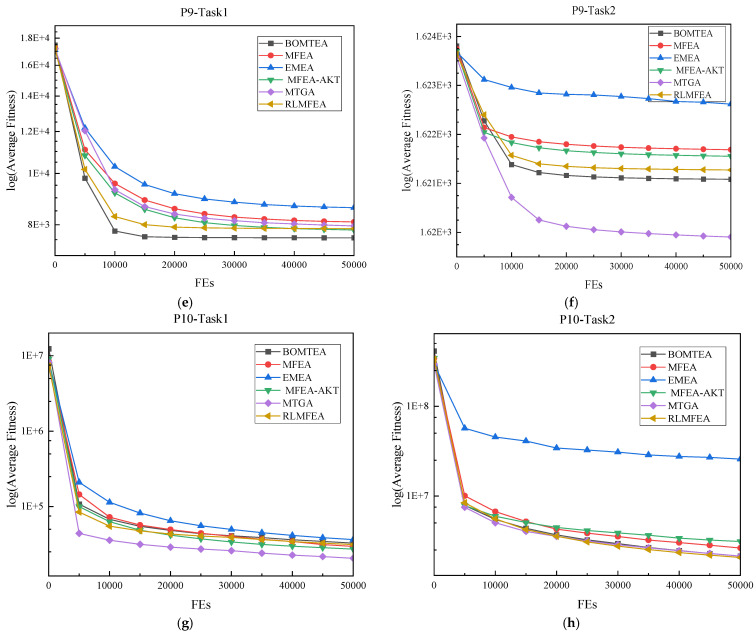
Convergence curves of the average fitness on (**a**) T1 of CEC22-P1; (**b**) T2 of CEC22-P1; (**c**) T1 of CEC22-P6; (**d**) T2 of CEC22-P6; (**e**) T1 of CEC22-P9; (**f**) T2 of CEC22-P9; (**g**) T1 of CEC22-P10; (**h**) T2 of CEC22-P10.

This article also presents the convergence curves of these algorithms on the CEC22 test set, as shown in Figure 2. Firstly, it can be seen from Figure 2 that RLMFEA and BOMTEA have similar convergence rates. Secondly, as shown in Figure 2d,e, although the convergence speed of BOMTEA on this problem is almost the same as that of RLMFEA in the early stage, the final results obtained by BOMTEA are superior to these two algorithms. Finally, as shown in Figure 2a,b, although BOMTEA initially converges at a rate similar to other algorithms, it is easy to fall into local optima in this question.

## 5. Conclusions

In this study, an innovative MTEA called MTEA via adaptive bi-operator (BOMTEA) is proposed. Compared to single operator algorithms, BOMTEA combines the superiority of GA and DE. Compared to other Bi-operator algorithms, the selection probability of each ESO is adaptively adjusted according to its performance, which can help algorithm find the suitable ESO in various tasks. Additionally, MTEA introduce a novel knowledge transfer strategy to enhance information sharing and communication across different tasks. The experimental results on the multitasking benchmarks CEC17 and CEC22 fully demonstrate the effectiveness of BOMTEA, which demonstrates a significant performance advantage over other comparative algorithms.

In the future, we wish to further explore the adaptive adjustment of *rmp* under the BOMTEA framework by introducing techniques such as fuzzy system or reinforcement learning. This will enable us to enhance the performance of the BOMTEA even further. In addition, we also wish to apply adaptive bi-operator strategy to multimodal [44,45,46] or large-scale [47,48,49] problems.

## Figures and Tables

**Table 1:** The CEC17 and CEC22 experimental results of BOMTEA and other EMTO algorithms.

Question	BOMTEA		MFEA		EMEA		MFEA-AKT		MTGA		RLMFEA	
	Task1	Task2	Task1	Task2	Task1	Task2	Task1	Task2	Task1	Task2	Task1	Task2
CIHS	**4.97e−04**	**4.78e+00**	3.80e−01 (+)	2.04e+02 (+)	5.66e−01 (+)	4.13e+02 (+)	3.42e−01 (+)	1.86e+02 (+)	2.72e−01 (+)	2.05e+02 (+)	1.89e−02 (+)	5.42e+01 (+)
CIMS	**3.69e−01**	**1.72e+01**	5.67e+00 (+)	2.71e+02 (+)	3.70e+00 (+)	4.14e+02 (+)	5.57e+00 (+)	2.54e+02 (+)	3.21e+00(+)	2.36e+02 (+)	2.32e+00 (+)	8.61e+01 (+)
CILS	2.01e+01	4.37e+03	2.02e+01 (+)	4.04e+03 (−)	2.05e+01 (+)	1.21e+04 (+)	2.02e+01 (+)	3.85e+03 (−)	**2.00e+01 (−)**	4.11e+03 (≈)	2.01e+01 (≈)	**3.24e+03 (−)**
PIHS	**2.01e+02**	**1.37e−03**	6.50e+02 (+)	1.18e+01 (+)	9.92e+02 (+)	3.43e−01 (+)	5.17e+02 (+)	9.07e+00 (+)	2.24e+02 (≈)	3.09e+00 (+)	2.37e+02 (+)	2.96e−02 (+)
PIMS	**3.48e−01**	**9.15e+01**	3.85e+00 (+)	8.16e+02 (+)	3.63e+00 (+)	3.19e+02 (+)	3.02e+00 (+)	3.74e+02 (+)	3.28e+00 (+)	5.14e+02 (+)	1.54e+00 (+)	1.35e+02 (+)
PILS	**1.42e+00**	2.13e+00	2.00e+01 (+)	2.16e+01 (+)	1.78e+01 (+)	**1.71e−01 (−)**	5.41e+00 (+)	5.81e+00 (+)	3.06e+00 (+)	5.72e+00 (+)	2.68e+00 (+)	3.21e+00 (+)
NIHS	**1.50e+02**	1.21e+02	7.68e+02 (+)	2.71e+02 (+)	6.37e+02 (+)	4.16e+02 (+)	5.18e+02 (+)	2.20e+02 (+)	5.68e+02 (+)	1.98e+02 (+)	2.12e+02 (+)	**1.16e+02 (≈)**
NIMS	**2.80e−03**	1.61e+01	4.17e−01 (+)	2.73e+01 (+)	7.31e−01 (+)	**1.20e+01 (−)**	4.13e−01 (+)	2.42e+01 (+)	3.79e−01 (+)	1.54e+01 (≈)	4.27e−02 (+)	1.99e+01 (+)
NILS	**2.04e+02**	4.33e+03	6.27e+02 (+)	3.77e+03 (−)	1.32e+03 (+)	1.21e+04 (+)	7.03e+02 (+)	3.90e+03 (−)	3.44e+02 (+)	4.36e+03 (≈)	2.89e+02 (+)	**3.23e+03 (−)**
P1	6.34e+02	6.34e+02	6.51e+02 (+)	6.53e+02 (+)	6.29e+02 (−)	**6.18e+02 (−)**	6.32e+02 (≈)	6.32e+02 (−)	**6.18e+02 (−)**	6.19e+02 (−)	6.30e+02 (−)	6.32e+02 (≈)
P2	**7.00e+02**	**7.00e+02**	7.01e+02 (+)	7.01e+02 (+)	7.05e+02 (+)	7.01e+02 (+)	7.01e+02 (+)	7.01e+02 (+)	7.01e+02 (+)	7.00e+02 (+)	7.00e+02 (+)	7.00e+02 (+)
P3	1.43e+06	1.59e+06	4.18e+06 (+)	3.63e+06 (+)	3.23e+06 (+)	6.33e+07 (+)	**1.22e+06 (≈)**	**1.25e+06 (≈)**	3.06e+06 (+)	2.84e+06 (+)	1.60e+06 (≈)	1.54e+06 (≈)
P4	1.30e+03	1.30e+03	1.30e+03 (+)	1.30e+03 (+)	1.30e+03 (+)	1.30e+03 (+)	1.30e+03 (+)	1.30e+03 (≈)	**1.30e+03 (−)**	**1.30e+03 (−)**	1.30e+03 (≈)	1.30e+03 (≈)
P5	**1.52e+03**	**1.52e+03**	1.56e+03 (+)	1.55e+03 (+)	1.79e+03 (+)	1.54e+03 (+)	1.56e+03 (+)	1.55e+03 (+)	1.53e+03 (+)	1.53e+03 (+)	1.53e+03 (+)	1.53e+03 (+)
P6	1.12e+06	**7.34e+05**	1.90e+06 (+)	1.60e+06 (+)	1.82e+06 (+)	2.66e+07 (+)	1.76e+06 (+)	1.57e+06 (+)	1.23e+06 (≈)	1.20e+06 (+)	**9.38e+05 (≈)**	9.66e+05 (≈)
P7	3.18e+03	3.19e+03	3.52e+03 (+)	3.52e+03 (+)	3.41e+03 (+)	4.64e+03 (+)	3.23e+03 (≈)	3.41e+03 (+)	**3.08e+03 (≈)**	**3.11e+03 (≈)**	3.19e+03 (≈)	3.20e+03 (≈)
P8	**5.20e+02**	**5.20e+02**	5.20e+02 (+)	5.20e+02 (+)	5.21e+02 (+)	5.21e+02 (+)	5.20e+02 (+)	5.20e+02 (+)	5.21e+02 (+)	5.21e+02 (+)	5.20e+02 (≈)	5.20e+02 (+)
P9	**7.56e+03**	1.62e+03	8.10e+03 (+)	1.62e+03 (+)	8.62e+03 (+)	1.62e+03 (+)	7.82e+03 (≈)	1.62e+03 (+)	7.96e+03 (+)	**1.62e+03 (−)**	7.87e+03 (≈)	1.62e+03 (≈)
P10	3.26e+04	2.14e+06	2.95e+04 (≈)	2.63e+06 (≈)	3.65e+04 (≈)	2.57e+07 (+)	2.72e+04 (≈)	3.11e+06 (+)	**2.05e+04 (−)**	2.14e+06 (≈)	3.12e+04 (≈)	**2.06e+06 (≈)**
Number of +/≈/−			18/1/0	16/1/2	17/1/1	16/0/3	14/5/0	14/2/3	12/3/4	11/5/3	10/8/1	9/8/2

## Data Availability

Data is contained within the article.
